# CancerOmicsStudio (CoS): a web server for integrative and interpretable analysis of multi-omics cancer data

**DOI:** 10.1093/bioinformatics/btag240

**Published:** 2026-05-20

**Authors:** Chenqi Liao, Rui Dou, Danyang Sun, Chengxiu Hu, Hui Hu, Long Cheng, Yuzhong Xu, Xiong Wang

**Affiliations:** Department of Laboratory Medicine, Tongji Hospital, Tongji Medical College, Huazhong University of Science and Technology, Wuhan, 430030, China; Institute of Hematology, Henan Key Laboratory of Stem Cell Differentiation and Modification, Henan Provincial People’s Hospital, People’s Hospital of Zhengzhou University, Zhengzhou, China; Department of Laboratory Medicine, Tongji Hospital, Tongji Medical College, Huazhong University of Science and Technology, Wuhan, 430030, China; Department of Laboratory Medicine, Tongji Hospital, Tongji Medical College, Huazhong University of Science and Technology, Wuhan, 430030, China; Department of Laboratory Medicine, Tongji Hospital, Tongji Medical College, Huazhong University of Science and Technology, Wuhan, 430030, China; Medical Affair Office, Tongji Hospital, Tongji Medical College, Huazhong University of Science and Technology, Wuhan, 430030, China; The Baoan People’s Hospital of Shenzhen, The Second Affiliated Hospital of Shenzhen University, Shenzhen, 518101, China; Department of Laboratory Medicine, Tongji Hospital, Tongji Medical College, Huazhong University of Science and Technology, Wuhan, 430030, China

## Abstract

**Motivation:**

Large-scale omics resources, including The Cancer Genome Atlas, Genomics of Drug Sensitivity in Cancer, and the Cancer Dependency Map, have become essential for cancer research. However, these datasets are distributed across different platforms, formats and analysis frameworks, which limits their practical use by researchers without extensive computational expertise.

**Results:**

We developed CancerOmicsStudio (CoS), a web server for integrative and interpretable analysis of multi-omics cancer data across 33 cancer types. CoS provides five major modules: CosAI, Traditional Analysis, Drug Sensitivity, CRISPR Dependency and Single-Cell Tumor Microenvironment. The Traditional Analysis module supports expression comparison, diagnostic evaluation, survival analysis, enrichment analysis and gene correlation. The Drug Sensitivity and CRISPR Dependency modules enable systematic evaluation of gene–drug response associations and gene essentiality in cancer cell lines. The Single-Cell Tumor Microenvironment module supports tumor microenvironment analysis at single-cell resolution. In total, approximately 1.23 million results have been precomputed to enable rapid retrieval. CosAI further allows users to submit natural-language queries and obtain results through a Real-time Analysis as Retrieval framework, with responses summarized by a lightweight language model.

**Availability and implementation:**

CancerOmicsStudio is freely available at Zenodo (doi: 10.5281/zenodo.18744990) and https://cos.wanglab.bio.

## 1 Introduction

Large-scale cancer genomics initiatives have generated extensive multi-omics datasets, including transcriptomic profiles, somatic mutations, pharmacogenomic responses, and functional dependency screens (Hanahan 2022, Swanton *et al.* 2024). Public portals have been developed to facilitate access to these resources. For bulk The Cancer Genome Atlas (TCGA) data, tools such as cBioPortal (Gao *et al.* 2013), GEPIA3 (Tang *et al.* 2017), and UCSC Xena (Goldman *et al.* 2020) provide interactive visualization of gene expression, mutation status, and survival associations across tumor types. These systems support hypothesis generation at the transcriptome and genomic levels and are widely used for pan-cancer exploration.

Pharmacogenomic resources, including Genomics of Drug Sensitivity in Cancer (GDSC) (Yang *et al.* 2013, Iorio *et al.* 2016) and PharmacoDB (Smirnov *et al.* 2018), compile drug-response measurements across cancer cell lines and enable association analyses between molecular features and therapeutic sensitivity. In parallel, the Cancer Dependency Map (DepMap) project (Chin *et al.* 2011, Das *et al.* 2020, Fan *et al.* 2020) systematically profiles gene essentiality using CRISPR-Cas9 screens, allowing identification of selectively vulnerable genes in specific cellular contexts.

More recently, single-cell atlases have provided cell-type-resolved characterization of the tumor microenvironment. Platforms such as TISCH2 (Han *et al.* 2023) and scCancerExplorer (Huang *et al.* 2025) provide organized access to tumor immune single-cell datasets and cell population-level expression profiles.

These platforms provide access to distinct data domains. TCGA-oriented portals primarily support bulk tumor-level transcriptomic and genomic analyses. Pharmacogenomic databases focus on cell-line drug response modeling. DepMap emphasizes gene essentiality profiling in vitro. Single-cell platforms provide cell-type-resolved expression patterns within the tumor microenvironment. However, most platforms are organized around a single data modality. Bulk transcriptomic analysis, drug sensitivity modeling, CRISPR dependency profiling, and single-cell exploration are typically performed through separate systems. Integrating results across these domains therefore requires cross-platform retrieval and manual harmonization.

CancerOmicsStudio (CoS) integrates TCGA bulk transcriptomic data, pharmacogenomic drug response datasets, CRISPR dependency screens, and tumor single-cell datasets within a unified analytical framework. Analyses are precomputed and indexed using a Real-time Analysis as Retrieval (RAR) strategy, enabling direct retrieval of cross-modal results without repeated computation. An optional AI-assisted module (CosAI) generates natural-language summaries while retaining the underlying statistical outputs. CoS is implemented as a retrieval-oriented framework that standardizes preprocessing across data types and supports integrative exploration without introducing new statistical methodologies.

## 2 Materials and methods

### 2.1 Data sources and preparation

The bulk TCGA datasets (gene expression (Colaprico *et al.* 2016), tumor mutation burden (TMB), microsatellite instability (MSI) (Ramos *et al.* 2020), DNA methylation profiles, immune cell proportions, immune scores (Yoshihara *et al.* 2013)) were downloaded and processed utilizing the TCGAplot R package (v8.0.0). Expression values were preprocessed as log_2_(TPM + 1) before downstream analysis. The processed datasets were saved in Parquet so that they could be quickly accessed through CoS (Liao and Wang 2023). In total, there are 11 235 TCGA samples (10 495 tumor, and 740 normal) representing 9678 unique patients from 33 cancer types, with expression values for 19 571 genes. All clinical variables (age, sex, stage, survival) were standardized prior to be integrated into our database.

Dose-response curves were downloaded from GDSC1/2 (release 27 Oct 2023). When available in both releases, we retained the release with more cell lines tested and opted for GDSC2 in case of ties (Yang *et al.* 2013, Iorio *et al.* 2016). After merging and deduplication, we obtained a dataset consisting of 756 cell lines with drug response and gene expression profiles corresponding to each of those 756 cell lines for modeling purposes, covering 30 cancer types (23 corresponding to TCGA tumor entities plus seven extra hematologic or pediatric cancer types). In total, we examined 621 compounds and utilized ln(IC50) and AUC as metrics for drug response (Barretina *et al.* 2012, Pozdeyev *et al.* 2016).

Gene dependency was retrieved from DepMap (Arafeh *et al.* 2025) (release 25 Q3) and computed via the CERES model that normalizes for copy-number-associated effects in CRISPR-Cas9 knockout screens (Meyers *et al.* 2017, Tsherniak *et al.* 2017). The dataset includes dependency scores for 18 159 genes across 1186 cell lines, classified according to the Oncotree primary disease. Non-cancer cell lines were included in pan-cancer analyses but excluded from cancer-type-specific summaries.

Single-cell transcriptomic data (TICAtlas, https://zenodo.org/records/5205544), spanning 342 758 cells from 13 tumor types, were downloaded and preprocessed in Scanpy with cell identities annotated according to hierarchical immune annotations comprising 25 immune cell subtypes (CD8^+^T, CD4+MemoryT, Macrophage, NK) (Wolf *et al.* 2018). Tumor classifications were grouped into 13 major cancer categories. Non-tumor or undefined samples were included in pan-cancer analyses and omitted from tumor-type-specific summaries.

### 2.2 CosAI and the real-time analysis as retrieval (RAR) architecture

CosAI functions as the primary query interface within CoS for hypothesis exploration. The Real-time Analysis as Retrieval (RAR) framework replaces on-demand computation with precomputed result retrieval through a three-layer structure: L1 (keyword-based indexing), L2 (precomputed pan-cancer analyses; ∼78 000 records), and L3 (cancer-specific analyses stratified by clinical subgroups; ∼1.15 million records). In total, the system indexes approximately ∼1.23 million analytical records to support query resolution. CosAI interfaces with a lightweight large language model (LLM) via the OpenRouter API to generate natural-language summaries from Python-derived analytical outputs, while preserving the underlying numerical results. CosAI supports single-gene as well as batch queries (multi-gene, multi-cancer, or pan-cancer combinations), which are resolved through the same precomputed retrieval framework.

### 2.3 Traditional analysis module

The Traditional Analysis module includes 28 statistical procedures organized into four categories: (1) Pan-Cancer Analysis provides expression profiling, paired tumor-normal comparisons, pan-tumor evaluations, and multi-cancer Cox regression. (2) Correlation & Immune Analysis examines associations with TMB and MSI and generates immune-related heatmaps for checkpoint, chemokine, and immune cell signatures. (3) Cancer-Specific Analysis supports subgroup-based differential expression (e.g., tumor-normal, age, gender, stage), DEG-driven GSEA, and co-expression analyses using GO and KEGG annotations. (4) Diagnostic & Survival Analysis incorporates ROC-based diagnostic assessment and Kaplan-Meier or Cox regression survival modeling. All analyses are implemented in Python (v3.10) with Pandas, StatsModels, GSEApy, and Plotly, and deployed through a Streamlit interface.

For ROC analysis, single-gene log_2_(TPM + 1) expression values from TCGA bulk RNA-seq data were used to discriminate tumor and adjacent normal samples within a selected cancer type. Tumor and normal samples were defined based on the TCGA “Group” annotation. ROC curves were computed using the sklearn implementation, and area under the curve (AUC) values were reported with 95% confidence intervals, which was estimated either via DeLong or bootstrapping (2000 iterations) depending upon whether sample size exceeded 5 tumor and 5 normal samples. For AUCs smaller than 0.5, direction was switched (AUC = 1 − AUC).

All gene-grouped analyses (DEG heatmap, GSEA, co-expression modules) were conducted on tumoral samples only. Samples were divided into two groups according to the median expression value of the chosen gene in the corresponding cancer type (high vs. low expression). Differential expression was tested via ordinary least squares (OLS) linear regression, modeling group (high/low expression) as a predictor. Raw P-values were calculated from t-statistics. Multiple testing correction was performed using the Benjamini-Hochberg false discovery rate (FDR) procedure. For visualization, the top N up- and down-regulated genes (default: 20 each) were displayed.

### 2.4 Drug sensitivity prediction

Drug response models were fitted using ridge regression (L2-regularized linear models) on GDSC expression-response data, separately for each of the 621 compounds. Model performance was evaluated using 5-fold cross-validation, with R^2^ and Pearson correlation between observed and predicted ln(IC50) values reported as performance metrics. For each compound, the 50 genes most strongly correlated with ln(IC50) values ranked by absolute value of the Spearman correlation coefficient were used as predictive features. The resulting models were applied to TCGA bulk expression data to estimate patient-level drug sensitivity in both pan-cancer and cancer-specific settings. Ridge regression coefficients were used to quantify feature contributions to predicted sensitivity. The module provides a summary view of drug-cell line associations, a gene correlation panel for pan-cancer and cancer-type analyses, a prediction panel reporting estimated sensitivities, and a drug recommendation panel ranking compounds according to predicted response. For subgroup comparisons of predicted drug sensitivity within tumor types, differences between high- and low-expression groups were evaluated using the Mann-Whitney U test.

### 2.5 CRISPR dependency analysis

Gene dependency data were obtained from DepMap (release 25Q3) and based on CERES scores. A gene was classified as pan-cancer essential if it showed strong dependency (CERES score < −0.5) in at least 50% of screened cancer cell lines. The pan-cancer essential genes were determined using this threshold on the dependency matrix without further re-definition.

Cancer-type-specific enrichment of gene dependency was determined with two-sided Fisher’s exact test comparing the proportion of dependent vs. non-dependent cell lines between tumor types. Genes were classified as pan-cancer essential or cancer-type–specific according to dependency distribution and statistical significance.

### 2.6 Single-cell tumor microenvironment analysis

Single-cell data from TICAtlas (342 758 cells across 13 cancer types) were analyzed with Scanpy (Nieto *et al.* 2021). We used cell type annotations provided in the original TICAtlas publication and did not perform further reannotation. This module allows for visualization of gene expression across pre-defined immune cell subtypes, comparison of immune cell composition across cancer types, and assessment of immune checkpoint gene expression in immune cell populations.

### 2.7 Web server implementation

Built with Streamlit (Python 3.10), the web caches analytical results using SQLite, stores bulk omics data in Parquet and H5AD formats, respectively, renders visualization components using Plotly that can be interactively filtered and exported figures, and has an AI-related component (CosAI) connected to the OpenRouter client to provide LLM-generated text.

## 3 Results

### 3.1 Platform overview

**Figure 1:**
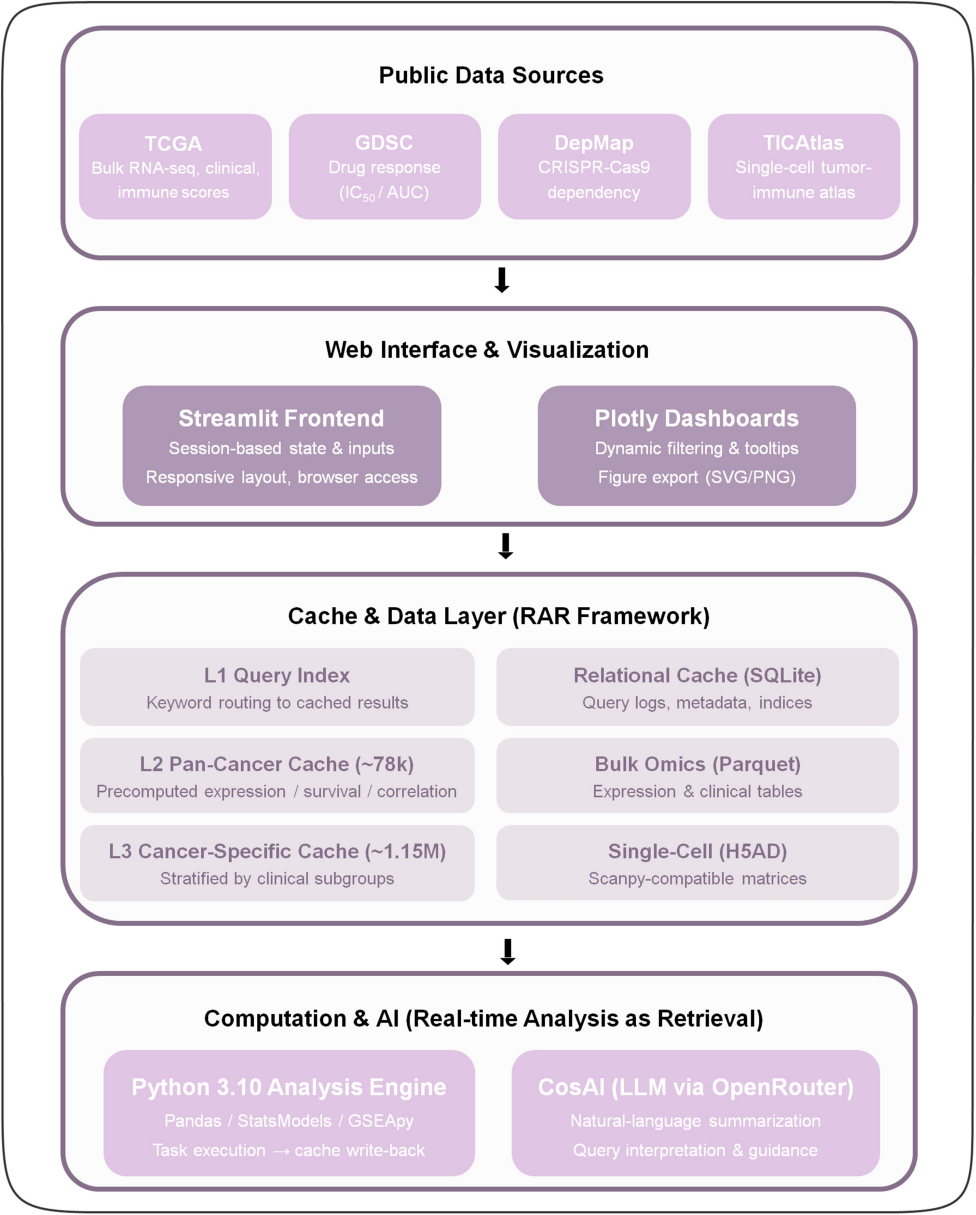
CancerOmicsStudio platform overview. CancerOmicsStudio (CoS) integrates multi-omics data from TCGA, GDSC, DepMap, and TICAtlas into five modules: CosAI, Traditional, Drug Sensitivity, CRISPR, and Single-Cell TME, enabling multi-level analyses, AI-assisted summarization, and interactive visualizations for therapeutic insights. The CoS system structure includes four parts: public data source layer, web UI layer (Streamlit + Plotly), cache and data storage layer (RAR Framework), computation and AI layer (Python engine + CosAI), which can be used for real-time analysis and AI-driven discovery of cancer-related information.

CancerOmicsStudio (CoS) supports multi-omics integration of 9678 TCGA patients, 621 anticancer drugs, 1186 CRISPR-screened cell lines, and 342 758 cells. The system groups bulk, pharmacogenomic, functional genomics, and single-cell data from four different perspectives of CancerOmicsStudio (CoS). The data were organized in the form of a four-layer framework based on the RAR model to achieve multi-scale and multi-dimensional data queries, analysis, and visualization. The analysis can be started from CoSAI and traverses four modules: Traditional Analysis, Drug Sensitivity Analysis, CRISPR Dependency Analysis, and Single-Cell TME Analysis. All four modules are connected in a unified pipeline to facilitate cross-scale and cross-modal exploration. It offers users interactive visualizations and AI-generated summaries showing links between drug response, gene dependency, and tumor microenvironment characteristics (Fig. 1).

### 3.2 CosAI: intelligent query and RAR caching

**Figure 2:**
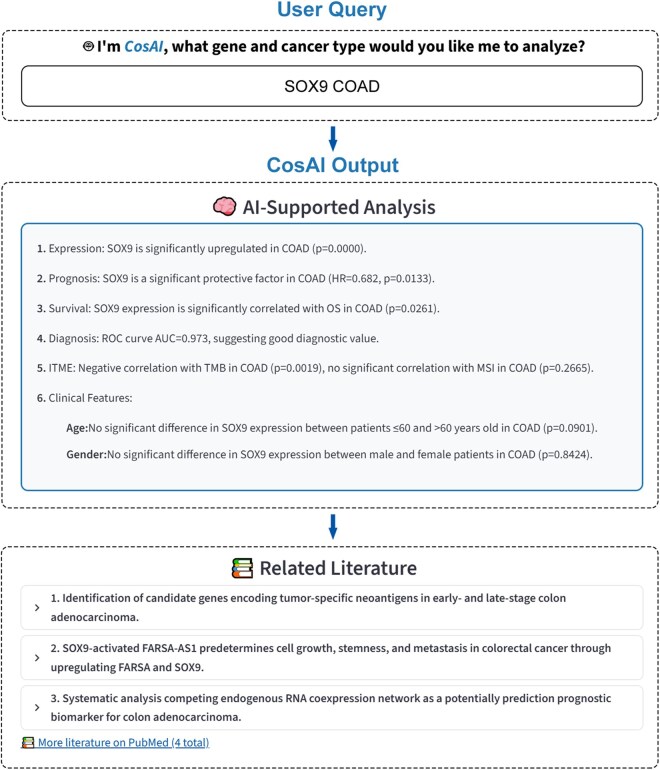
CosAI workflow for intelligent queries and automated analyses. CosAI supports rapid gene-cancer studies with a three-step workflow. (Top) User Query: Enter gene(s) and cancer type(s) (e.g., “SOX9 COAD”). (Middle) CosAI Output: Precomputed multi-omics results (expression, prognosis, survival, immune correlations (TMB/MSI), comparisons). (Bottom) PubMed Literature: Literature search results provide biological context and help formulate hypotheses.

CosAI provides keyword-based or structured batch queries. Users can perform single gene/single cancer queries (e.g., “SOX9 COAD”) or multi-gene/multi-cancer, pan-cancer, or matrix-style queries (e.g., “SOX9, ERBB2 COAD, READ” or “SOX9 PAN-CANCER”). CosAI retrieves precomputed analytical results from the RAR database for every gene/cancer pair and returns structured summaries regarding expression, prognosis, diagnostic performance, and immune associations. Batch queries are processed sequentially using the same retrieval framework and presented together. The RAR architecture ensures that all results are derived from precomputed statistical analyses rather than real-time computation, while the embedded LLM is used exclusively for text summarization (Fig. 2).

### 3.3 Traditional analysis: expression, survival, and correlation

**Figure 3:**
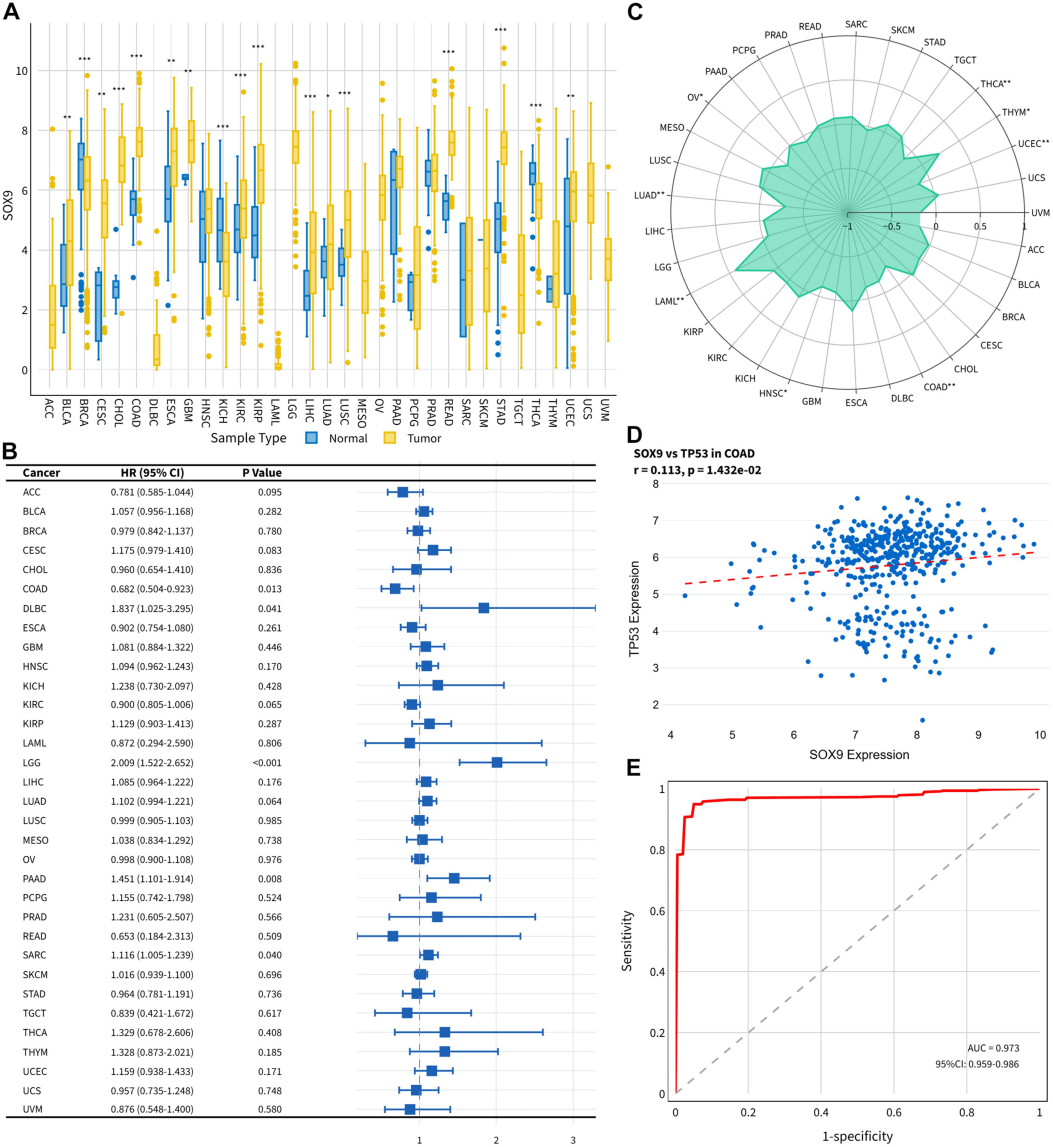
Pan-cancer and COAD specific multi-omics analyses of SOX9. (A) Differential expression of SOX9 in 24 TCGA cancer types. (B) Pan-cancer Cox analysis of SOX9 expression. (C) SOX9-TMB correlation analysis. (D) Correlation between SOX9 and TP53 expression in COAD (*r* = 0.113, *p* = 1.43e-02). (E) ROC analysis of SOX9 expression in COAD.

The Traditional Analysis module provides pan-cancer comparisons, correlation profiling, clinical subgroup analyses, and survival modeling. SOX9 showed significant tumor-normal differences in 17 of 24 evaluable TCGA cancers, including 14 upregulated (e.g., COAD, LUAD, LUSC, STAD) and 3 downregulated (BRCA, KICH, THCA) types (Fig. 3A). Cox analysis indicated that SOX9-high expression was protective in COAD, but deleterious in SARC, PAAD, LGG, and DLBC (Fig. 3B). Gene-TMB correlation showed positive associations with TMB in THYM and LAML, and negative associations in UCEC, THCA, OV, LUAD, HNSC, and COAD (*p* < 0.05) (Fig. 3C). In COAD, SOX9 showed a weak positive correlation with TP53 (*r* = 0.1126, *p* = 1.43e-02) (Fig. 3D). ROC analysis showed excellent discriminatory performance with AUC = 0.9726 (95% CI: 0.9594–0.9859) (Fig. 3E).

### 3.4 Drug sensitivity: gene-drug correlations and patient-level prediction

**Figure 4:**
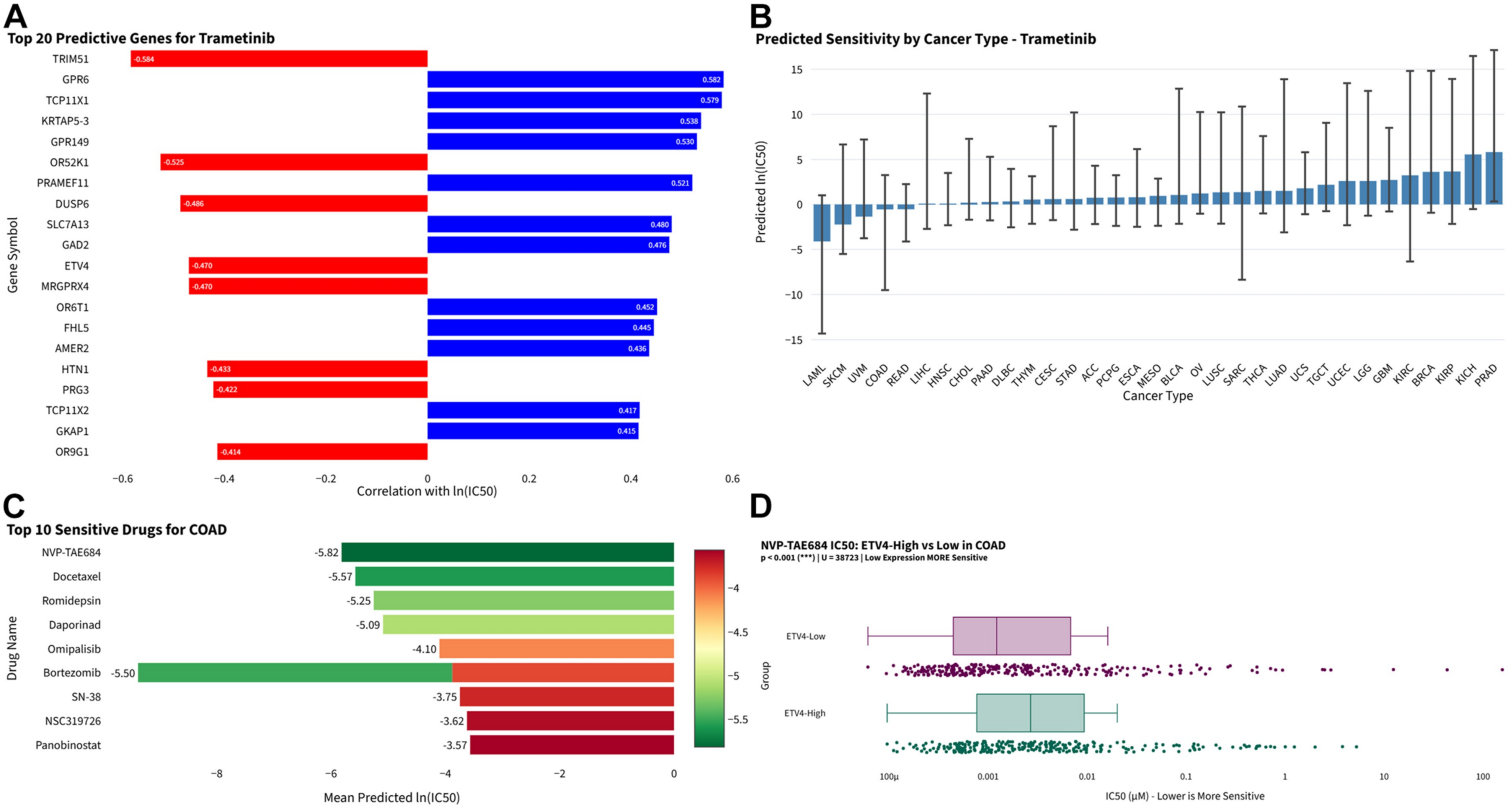
Drug sensitivity analysis and patient-level prediction. (A) Gene-drug correlation analysis for Trametinib (GDSC2), showing top predictive genes ranked by correlation with ln(IC_50_). (B) TCGA-wide Trametinib sensitivity predictions. (C) Cancer-type specific drug recommendations for COAD illustrating top-ranked agents. (D) Comparison of predicted NVP-TAE684 sensitivity between ETV4-high and ETV4-low COAD tumors.

The association between gene expression and drug response was examined by integrating GDSC dose-response profiles with TCGA transcriptomic data. Using Trametinib (GDSC2; MEK inhibitor) as an example, expression of TRIM51 was strongly negatively correlated with ln(IC_50_) (*ρ* = −0.584, *p* = 1.9 × 10^−14^), together with several MAPK pathway-related genes, including DUSP6 and ETV4 (Fig. 4A). Ridge regression models built on the top 50 predictive genes achieved a cross-validated R^2^ of 0.405 ± 0.085. Applying these models to TCGA samples yielded predicted sensitivities across 14 cancer types and revealed substantial inter-tumor variability. LAML had the lowest predicted ln(IC_50_), consistent with higher sensitivity, whereas PRAD and KICH had higher values, indicative of the relatively lower sensitivity (Fig. 4B). In COAD, NVP-TAE684, Docetaxel, Bortezomib, Romidepsin, and Daporinad were predicted to be the most sensitive drugs by mean predicted ln(IC_50_); however, Trametinib had a predicted ln(IC_50_) that was significantly higher than several other agents in COAD (Fig. 4C). Finally, we investigated if gene expression profiles correlate with variations in predicted drug response among different subgroups of patients per tumor type. Samples were divided into two groups according to the median expression level of the corresponding gene, and predicted ln(IC_50_) scores were compared between the high- and low-expression groups via Mann-Whitney U test. For instance, in COAD, the predicted ln(IC_50_) values for NVP-TAE684 were significantly lower in patients with low ETV4 expression versus high expression (*p* = 7.101 × 10^−4^; Fig. 4D). This implies that there may be differential sensitivity towards NVP-TAE684 in the ETV4-low vs. high groups of COAD patients. Similarly, other predicted responses were also consistent with previously reported pathway activities in various cancer types.

### 3.5 CRISPR dependency analysis

Across 1186 cell lines, CRISPR screens revealed both essential and context-dependent genes. 143 genes had dependency scores under −2.0 in all cell lines, signifying highly and broadly essential genes. Genes involved in gene expression control, protein quality control, cell division, and DNA replication were classified into functional groups (ribosome, proteasome, RNA splicing, RNA polymerase, cell cycle, molecular chaperone, DNA replication, protein trafficking, and ubiquitination).

**Figure 5:**
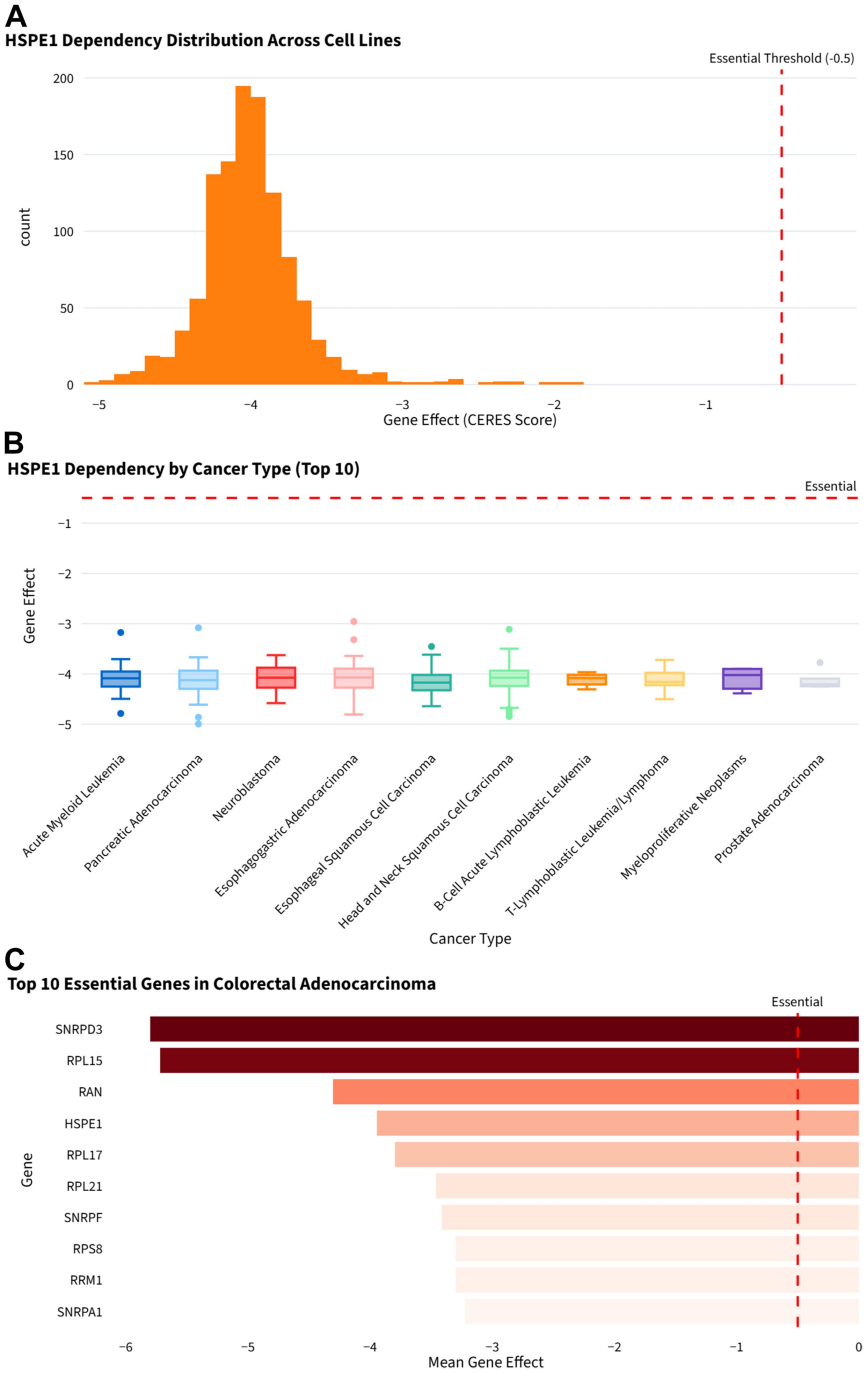
CRISPR dependency analysis. (A) HSPE1 dependency distribution. (B) HSPE1 dependency analysis by cancer type. (C) Top 10 essential genes in Colorectal Adenocarcinoma.

HSPE1 had consistently dependent properties in various cancers, with a mean gene effect of −4.003 and minimal gene effect of −5.015 (Fig. 5A). HSPE1 had the highest dependency in Pancreatic adenocarcinoma, Colorectal adenocarcinoma, and Head and Neck squamous cell carcinoma (Fig. 5B). In Colorectal Adenocarcinoma, HSPE1 showed a dependency score of −3.944 and ranked among the top essential genes. Additional essential genes in Colorectal Adenocarcinoma included SNRPD3, RPL15, RAN, and RPL17 (Fig. 5C).

### 3.6 Single-Cell Tumor microenvironment

**Figure 6:**
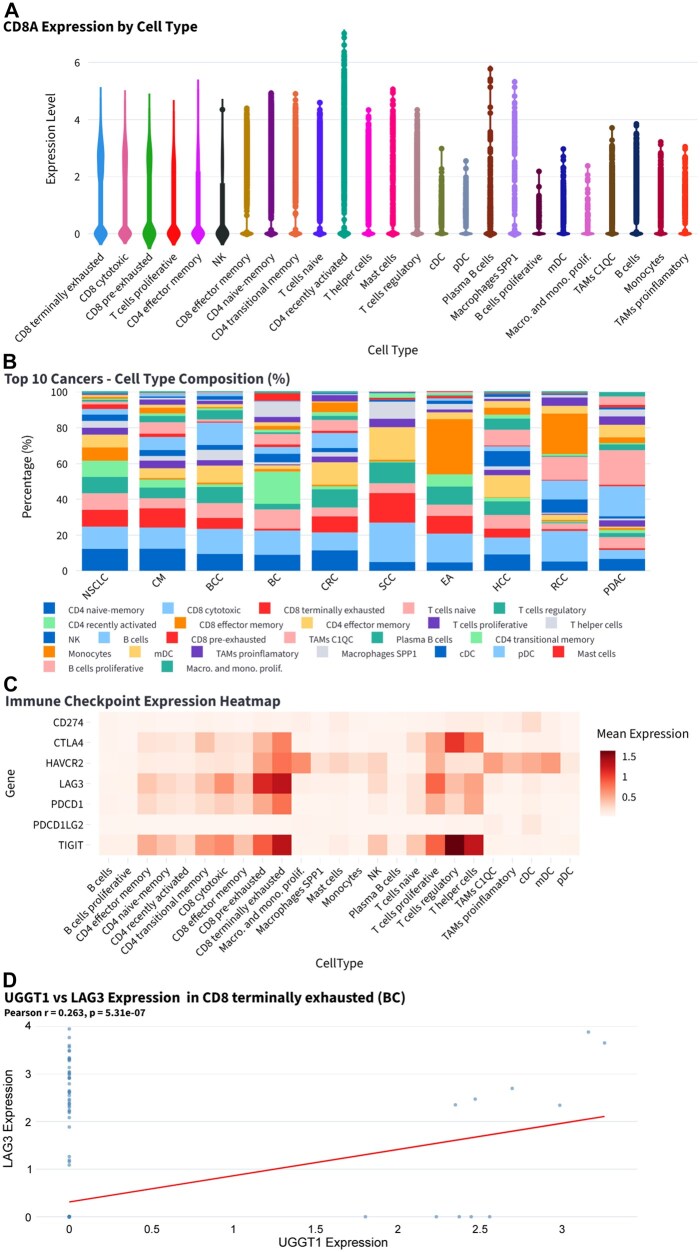
Immune cell composition and immune checkpoint expression. (A) Expression levels of CD8A across immune cell types in all cancer types. (B) Cell composition by cancer type. (C) Expression of immune checkpoint genes across various immune cell types. (D) Gene correlation analysis between UGGT1 and the immune checkpoint gene LAG3 in CD8 terminally exhausted cells in BC.

The Single-Cell Tumor Microenvironment (TME) module analyzes 342,758 cells across 25 cell types and compares immune cell composition among cancer types. CD8A, a marker of CD8 T cells, is enriched in terminally exhausted and cytotoxic CD8 populations (Fig. 6A). SCC and RCC show higher proportions of CD8 cytotoxic cells, whereas HCC and PDAC display lower immune cell infiltration (Fig. 6B). PDCD1 expression is elevated in exhausted CD8 T cells (Fig. 6C). Gene correlation analysis shows an association between UGGT1 and LAG3 in CD8 terminally exhausted cells in BC (Fig. 6D).

### 3.7 Integrated multi-module workflow

CancerOmicsStudio integrates multiple analytical modules, illustrated using ERBB2 (HER2) in Breast Cancer (BRCA) (Krishnamurti and Silverman 2014), an FDA-approved therapeutic target (Ruiz-Saenz *et al.* 2018). ERBB2 is overexpressed in BRCA tumors (log_2_FC = 0.24, *p* < 0.001, *n* = 1113). Drug sensitivity models predict that 307 of 1,226 BRCA patients (25.0%) were predicted to be sensitive to Lapatinib, an FDA-approved EGFR/HER2 inhibitor (Yuan *et al.* 2022). CRISPR screening shows that 32.7% of invasive breast carcinoma cell lines (*n* = 49) exhibit ERBB2 dependency. This workflow links gene expression, drug response, and functional dependency within a unified analysis framework.

### 3.8 Case study: integrated analysis of SOX9 in COAD

To illustrate cross-module integration, we examined SOX9 in COAD. Consistent with the pan-cancer results, SOX9 was upregulated in tumors and associated with favorable overall survival. SOX9 expression showed a negative correlation with TMB and weak negative correlations were observed with CD274 (*r* = −0.130), IFNG (*r* = −0.101), and CXCL10 (*r* = −0.094).

Drug response prediction across 514 TCGA COAD samples identified NVP-TAE684, Docetaxel, and Bortezomib among agents with low predicted ln(IC_50_) values. Stratification by SOX9 expression revealed differential sensitivity: NVP-TAE684 and Docetaxel showed lower predicted ln(IC_50_) in SOX9-low tumors (*p* < 1 × 10^−4^), whereas Bortezomib showed greater sensitivity in SOX9-high tumors (*p* = 1.241 × 10^−4^).

In CRISPR screens (*n* = 63 COAD cell lines), SOX9 had a mean gene effect of −0.580 (*SD* = 0.476; minimum −2.060). Strong dependency (effect < −1) was observed in 20.6% of models, and 52.4% fell below the −0.5 essentiality threshold. SOX9 ranked 1515/17,679 genes in COAD (top 9%), indicating substantial but context-dependent dependency.

### 3.9 Systematic comparison with existing cancer multi-omics platforms

We performed a comparison between CoS and several commonly used cancer platforms, including cBioPortal, GEPIA3, TIMER3, PharmacoDB, and DepMap (Table 1). The comparison included data integration scope, analytical functions, computational architecture and response time, and AI-assisted interpretation. CoS integrates TCGA bulk RNA-seq, pan-cancer survival data, GDSC drug sensitivity data, CRISPR dependency data, and single-cell tumor microenvironment datasets in one system. The other platforms mainly focus on specific data types. CoS provides pan-cancer Cox regression, ROC analysis, gene-TMB and gene-MSI correlations, co-expression with enrichment analysis, and context-specific CRISPR essentiality. These functions are not simultaneously available in the compared tools. CoS is based on a precomputed RAR framework with a multi-layer cache system, while most other platforms use real-time computation. In testing across 20 representative gene-cancer queries, the median end-to-end response time was 2.8 s (IQR 2.4–3.3) under standard network conditions. The AI-assisted module CosAI generates structured summaries from precomputed statistical results using fixed templates. Manual checking showed that the reported statistics were consistent with the underlying database values.

**Table 1:** Systematic comparison.

Feature	CoS	cBioPortal	GEPIA3	TIMER3	PharmacoDB	DepMap
*Section 1: Data Integration Scope*						
TCGA bulk RNA-seq	✓	✓	✓	✓	✗	✗
Pan-cancer survival analysis	✓	✓	✓	✗	✗	✗
Tumor-normal comparison	✓	Partial	✓	✓	✗	✗
Drug sensitivity integration (GDSC)	✓	✗	✗	✗	✓	Partial
Patient-level drug response prediction	✓	✗	✗	✗	✗	✗
CRISPR dependency integration	✓	✗	✗	✗	✗	✓
Single-cell tumor microenvironment	✓	✗	✗	✗	✗	✗
Cross-modal linking	✓	✗	✗	✗	✗	✗
*Section 2: Analytical Function Coverage*						
Pan-cancer Cox regression profiling	✓	✓	Partial	✗	✗	✗
ROC analysis	✓	✗	✓	Partial	✗	✗
Gene-TMB correlation	✓	✗	✗	✗	✗	✗
Gene-MSI correlation	✓	✗	✗	✗	✗	✗
Co-expression heatmap + enrichment	✓	✗	✗	✗	✗	✗
Context-specific CRISPR essentiality	✓	✗	✗	✗	✗	✓
Integrated workflow across modules	✓	✗	✗	✗	✗	✗
*Section 3: Computational Architecture & Data Processing Speed*						
Real-time computation	✗	✓	✓	✓	✓	✓
Precomputed retrieval architecture (RAR)	✓	✗	✗	✗	✗	✗
Multi-layer cache system	✓	✗	✗	✗	✗	✗
Average query response time (seconds)	2.8 s	Variable	Variable	Variable	Variable	Variable
*Section 4: AI-Assisted Interpretation*						
LLM-generated structured summaries	✓	✗	✗	✗	✗	✗
Template-constrained output	✓	✗	✗	✗	✗	✗
Numerical consistency validation	✓	✗	✗	✗	✗	✗

## 4 Discussion

CoS integrates multiple data resources for multi-omics cancer analysis. The RAR framework precomputes ∼1.23 million analytical records and replaces on-demand computation with cached retrieval. The system incorporates datasets from TCGA, GDSC, DepMap, and TICAtlas to support analyses across gene expression, drug response, CRISPR dependency, and single-cell data (Le *et al.* 2025, Park *et al.* 2025). CosAI uses a lightweight LLM to generate natural-language summaries of analytical outputs.

Planned updates include integration of proteomic and metabolomic datasets, support for user-uploaded data, and development of a public API. Molecular docking functionality is under development for evaluating small-molecule interactions with selected gene targets (Mohs and Greig 2017). Multi-gene analysis at the gene set level is also planned. The platform will continue to incorporate updated releases from TCGA, GDSC, and DepMap.

Recent studies have increasingly highlighted the value of integrative transcriptomic and multi-omics strategies for systematic characterization of the tumor microenvironment, prognostic biomarker discovery, and drug sensitivity prediction. These approaches can support systematic immune microenvironment assessment and help elucidate treatment sensitivity or resistance mechanisms across cancer types. Representative studies have shown that integrated analysis frameworks can improve the interpretation of tumor microenvironmental features and antitumor immunity (Fang *et al.* 2025), while transcriptome-based analyses in lung cancer and glioma further illustrate the utility of such strategies for drug response interpretation and prognosis assessment (Chen *et al.* 2020, Wang *et al.* 2020, Zeng *et al.* 2021). Together, these findings suggest that gene expression data can reveal not only tumor-intrinsic biology but also immune contexture and therapy-related features. In this context, CoS provides a unified and user-friendly platform that connects gene expression, immune features, drug sensitivity, CRISPR dependency, and single-cell tumor microenvironment analyses, thereby enabling interpretable and hypothesis-generating exploration of tumor-immune-therapy relationships. By integrating transcriptomic profiling with drug sensitivity and functional dependency information, CoS may facilitate the identification of context-specific vulnerabilities and support a more comprehensive understanding of complex cancer biology.

The drug sensitivity module is based on ridge regression models trained on GDSC cell line data using the top 50 correlated genes per drug. Several limitations should be acknowledged. First, we did not systematically benchmark alternative machine learning algorithms. Second, model performance varies across drugs, and predictions are derived from in vitro cell line systems rather than independent clinical response cohorts. Third, transferring models from cell lines to bulk tumor transcriptomes may be influenced by tumor purity and microenvironmental heterogeneity. Prediction results were not validated independently. Therefore, predicted sensitivity values can be considered exploratory/hypothesis-generating and not directly indicative of clinical response/precision treatment. Finally, CoS currently runs on curated publicly available datasets, does not accept user-uploaded data, and thus analysis is limited to the integrated reference cohorts within the tool. User provided data upload capabilities are planned for future work to expand application to primary research.

To make it more convenient for non-experts to use, CoS has developed an “Examples” webpage displaying sample input values and typical output results from every module. Core datasets (TCGA, GDSC, DepMap, etc.) are refreshed according to authoritative updates, and corresponding versions are logged on the system. By adopting the RAR model we lower the cost of computation during runtime, ensure reliable operation when multiple people request data simultaneously, schedule regular checks to guarantee uninterrupted service.

## Data Availability

CancerOmicsStudio (CoS) is publicly accessible at https://cos.wanglab.bio/ without login requirements. The source code and precomputed analytical scripts are archived on Zenodo (doi: 10.5281/zenodo.18744990). All integrated datasets are publicly available from their original sources, including TCGA (https://portal.gdc.cancer.gov/), GDSC (https://www.cancerrxgene.org/), DepMap (https://depmap.org/portal/), and TICAtlas (https://zenodo.org/records/5205544).
